# The Effectiveness and Safety of Stem Cell-Based Tissue Engineering in the Regeneration of Periodontal Bone Lesions: A Systematic Review

**DOI:** 10.3390/clinpract15120222

**Published:** 2025-11-26

**Authors:** Marouan Fanid, Ana Sofia Vinhas, Cátia Reis, Marta Relvas, Rosana Costa, Cristina Cabral

**Affiliations:** 1 Faculty of Dental Medicine, University Institute of Health Sciences (IUCS), Cooperativa de Ensino Superior Politécnico e Universitário (CESPU), 4585-116 Gandra, Portugal; 2 Department of Medicine and Oral Surgery, University Institute of Health Sciences (IUCS-CESPU), 4585-116 Gandra, Portugal; ana.vinhas@iucs.cespu.pt (A.S.V.); catia.reis@iucs.cespu.pt (C.R.); marta.relvas@iucs.cespu.pt (M.R.); rosana.costa@iucs.cespu.pt (R.C.); cristina.cabral@iucs.cespu.pt (C.C.); 3 Oral Pathology and Rehabilitation Research Unit (UNIPRO), University Institute of Health Sciences (IUCS-CESPU), 4585-116 Gandra, Portugal

**Keywords:** periodontal defects, alveolar bone regeneration, tissue engineering, cell transplantation, stem cell therapy

## Abstract

**Background/Objectives:** Periodontal diseases are highly prevalent worldwide, causing progressive destruction of the alveolar bone and eventual tooth loss when not treated. Despite advances in conventional periodontal therapies, complete tissue regeneration remains limited. This review aims to evaluate the efficacy, safety, and clinical relevance of stem cell-based tissue engineering approaches for regeneration of periodontal bone lesions. **Methods:** Following PRISMA guidelines, a systematic search was conducted across multiple databases, resulting in the inclusion of 17 studies in humans that met predefined PICO criteria. The study protocol was registered on PROSPERO (CRD420251229271). These studies assessed various stem cell sources, including dental and bone marrow-derived cells among others, both on their own and in combination with scaffolds or growth factors. **Results:** Most studies reported favorable outcomes in terms of clinical attachment gain, radiographic bone fill, probing depth reduction, and implant stability. No major adverse effects were noted, indicating good safety. However, results varied based on cell type, culture protocols, and defect characteristics. **Conclusions:** Stem cell therapy shows strong potential for periodontal regeneration, with outcomes that may potentially surpass those of conventional methods in selected cases. Further standardization, cost reduction, and long-term clinical trials are essential to confirm these findings and support their integration into daily dental practice.

## 1. Introduction

Periodontal diseases, including gingivitis and periodontitis, are among the most prevalent chronic conditions affecting the global adult population. These diseases affect approximately 20% to 50% of individuals worldwide, representing over one billion people [1]. Characterized by localized inflammation of the periodontal tissues that support the teeth, periodontal diseases initially manifest with edema, swelling, and bleeding of the gums, and can progress to alveolar bone and periodontal ligament destruction [2].

There are clear geographical and demographic disparities in the distribution of these diseases. Some studies attribute this phenomenon to the impact of contemporary industrial diets [3].

If untreated, periodontal disease can cause tooth loss and impair oral [4], mental, and social [5] well-being.

Though usually slow, periodontal disease can worsen with genetic predisposition [4], systemic disorders [6,7,8,9,10,11], and other factors like smoking and obesity [12,13,14].

The principal cause remains poor oral hygiene, which promotes plaque accumulation, leading to inflammation. Bacterial enzymes and cytokines such as IL-1 and TNF-α, play key roles in tissue destruction [15].

Periodontal disease is classified using standardized criteria [16]; conventional treatments like scaling, root planing and mucogingival surgery [2] help to control progression, restore health and esthetics, but they cannot regenerate lost tissues.

Regeneration of periodontal tissues remains a major challenge in dental medicine but holds considerable promise for public health. Given to the complexity of bone regeneration mechanisms, advanced research is needed. Recently, stem cell-based therapies have shown promising potential [17].

Stem cells may be obtained from different tissues, such as embryonic, mesenchymal, and dental sources. Dental-derived stem cells include dental pulp stem cells (DPSCs), gingival mesenchymal stem cells (GMSCs), and periodontal ligament stem cells (PDLSCs) [18]. These cells possess the ability to differentiate into osteoblasts, thereby contributing to the regeneration of periodontal lesions and enhancing bone quality, particularly in the context of dental implant placement. When combined with growth factors and biomaterials, stem cells have become key elements in tissue engineering and guided tissue regeneration strategies [17]. Given their proliferative and differentiation capacities, stem cells are notable for their strong regenerative potential. For this reason, they are considered one of the most promising regenerative methods in the field of periodontal therapy. Recent studies evaluating their safety, efficacy, and clinical applicability have produced encouraging results, offering a hopeful perspective for the future and paving the way for the broader adoption of these treatments in routine dental practice. Although the current body of research remains relatively limited, the findings are highly promising and offer genuine hope for improved management of periodontal diseases.

### Objectives

The importance of this study lies in the growing demand for effective periodontal treatments. Periodontal regeneration remains a major challenge, as the implementation of innovative therapeutic approaches could bring new perspectives and improve oral health outcomes. A comprehensive review of recent advancements is therefore essential to assess the current state of research and identify potential clinical applications. This study aims to evaluate whether stem cell therapy represents an effective and safe approach for treating periodontal bone lesions compared with established therapeutic methods.

## 2. Materials and Methods

The systematic review was initiated in January 2025 with bibliographic searches and determination of the subject and objectives. It was concluded in October 2025, following the guidelines of the “Preferred Reporting Items for Systematic Reviews and Meta-Analyses guidelines” (PRISMA) [19], through the databases MEDLINE via PubMed, Cochrane Library, Web of Science, and SciELO. The PRISMA checklist is provided as Supplementary Material. The PRISMA 2020 checklist, which provides a detailed overview of the reporting standards followed in this review, can be consulted in the Supplementary Materials.

### 2.1. PICO

The initial screening of studies was performed using the title, abstract, and full text of the selected studies, by two independent investigators (Student and academic advisor). Studies were considered for inclusion only if they satisfied all predefined criteria outlined by the PICOS framework (Population, Intervention, Comparison, Outcomes, and Study design).

Eligibility criteria were defined using the PICO framework, as outlined below:

- P (Population): Adult patients presenting with periodontal bone lesions.
- I (Intervention): Stem cell-based tissue engineering therapies.
- C (Comparison): Stem cell-based therapies compared with alternative techniques.
- O (Outcome): Evaluation of the efficacy of current stem cell-based approaches.

**Figure 1:**
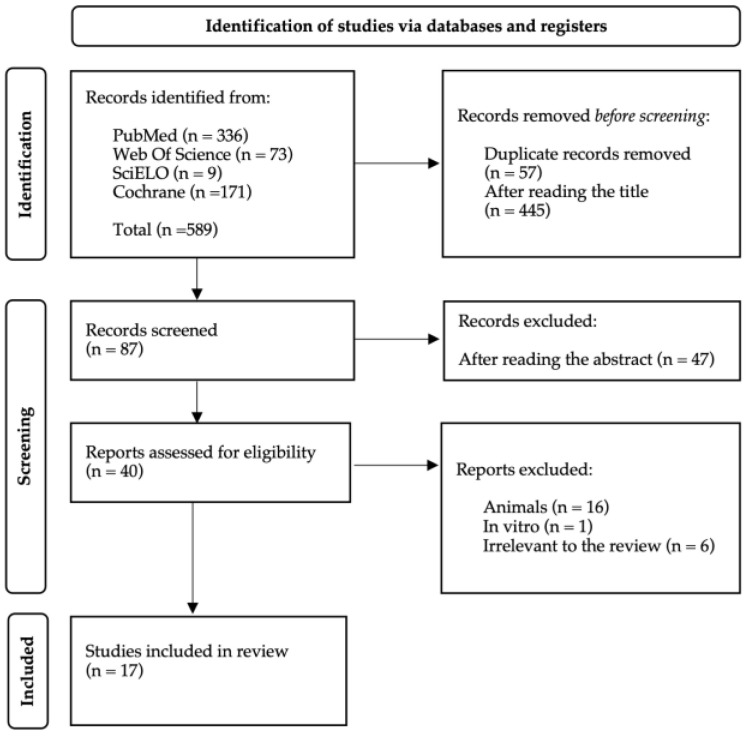
PRISMA flowchart summarizing the study identification and selection process.

The Results section presents a detailed flowchart of the search process (Figure 1).

The study protocol for this systematic review was registered on the International Prospective of Systematic Reviews (PROSPERO), under number CRD420251229271.

### 2.2. Research Strategy

The research strategy used was: MeSH Terms: (periodontal disease[MeSH Terms]) AND (stem cells [MeSH Terms]); (periodontitis [MeSH Terms]) OR (periodontal attachment loss [MeSH Terms]) AND (stem cells [MeSH Terms]); (periodontal disease [MeSH Terms]) AND (Tissue engineering [MeSH Terms]) OR (guided tissue regeneration [MeSH Term]); (stem cells [MeSH Terms]) AND (Bone regeneration [MeSH Terms]).

### 2.3. Eligibility Criteria

The inclusion criteria based on the PICO questions focused on alveolar bone stem cells therapies, and clinical studies, clinical trials (Protocol, Phase I/II/III/I), Randomized, Adaptive, Controlled or not, case reports, classical article, multicenter study and observational study, within the period from 2020 to 2025.

Articles without accessible abstracts, systematic reviews, meta-analyses, animal and In Vitro studies, and studies on other bone regeneration or regenerative therapies. Additionally, we excluded articles related to treatments that could alter study parameters, such as drugs or systemic diseases that could affect the progression of periodontal disease or response to treatment.

### 2.4. Extraction of Sample Data

Table 1 presents the information collected, considering the authors, title (year), study design and aim, study population (including sample size, age range and mean age, teeth identification, sex), type of stem cell therapy and possible adjuvant therapy, outcome measures, and results. Information is organized according to the year of publication, from most recent to least recent (Table 1).

### 2.5. Assessment of Publication Bias

Assessment of publication bias through funnel plots and statistical tests (Egger, Begg) was not feasible due to insufficient studies per clinical indication, heterogeneous study designs and outcome measures.

### 2.6. Study Quality and Risk of Bias

To evaluate the methodological quality of the included studies and determine how effectively they addressed potential sources of bias in their design, conduct, and analysis, we followed the Joanna Briggs Institute (JBI) 2017 guidelines, applying the appropriate checklist for each study design (case reports and randomized controlled trials) [20]. For each study type, a specific questionnaire was applied, offering four possible responses: Yes (Y), No (N), Unclear (UN), and Not Applicable (NA). Two reviewers (M.F. and C.C.) independently assessed each article for inclusion, data extraction, and quality evaluation, thereby ensuring intra- and inter-examiner reliability. In case of disagreement, a discussion was undertaken until consensus was reached. If no consensus could be achieved, a third reviewer (M.R.) acted as an arbiter to make the final decision.

## 3. Results

### 3.1. Search Strategy

In total, 589 studies were initially identified. Duplicates were removed, and studies were excluded based on title and abstract screening. Studies conducted in non-human subjects were also excluded. The final review comprised 17 studies, all of which underwent full-text analysis, as depicted in Figure 1.

A total of 17 studies were included in the review; the characteristics of these studies are presented in Table 1.

### 3.2. Characterization of the Included Studies

Based on our findings, we identified 10 randomized controlled trials [21,22,23,24,25,26,27,28,29,30] and 7 case reports [31,32,33,34,35,36,37]. According to subject, 9 studies focused on periodontal intrabony defects [21,22,23,24,25,27,28,29,30], 2 studies evaluated dental implant stability or osseointegration [26,35], 5 focused on regenerative endodontics for the treatment of apical periodontitis [31,32,33,34,37].

Although randomized controlled trials provide a higher level of evidence, there is still a limited number of RCTs focusing on stem cell-based tissue engineering. To provide a review of the literature and to consider clinical feedback, we included both randomized trials and recent case reports.

**Table 1:** The main characteristics of the included studies.

Author, Year of Publication	Title	Study Design	Study Aim	Type of Cellular Therapy	Sample Size	Outcome measures	Conclusion
Sreeparvathy et al., 2024 [21]	“Platelet Rich Fibrin Matrix (PRFM) and Peripheral Blood Mesenchymal Stem Cells (PBMSCs) in the management of intraosseous defects—A randomized clinical trial”	Randomized Controlled trial	“Evaluate the regenerative capacity of supercell (PRFM and PBMSCs) compared with that of platelet rich fibrin matrix (PRFM) alone in human periodontal mandibular intraosseous defects”	“Supercell (PRFM and PBMSCs) compared with that of PRFM”	“17 patients of both sexes (12 male, 5 female) Age [30;55] years $\bar{x}$ = (37.7 ± 4.4 years)”	“- Plaque index (PI), - Gingival index (GI) - Probing pocket depth (PPD) - Clinical attachment level (CAL) - Defect depth (DD) - Defect fill percentage (DFP)”	“Supercell can be considered a regenerative material in the treatment of periodontal IODs.”
Apatzidou et al., 2024 [22]	“Inflammatory and bone remodeling related biomarkers following periodontal transplantation of the tissue engineered bio complex”	Randomized Clinical trial	“To assess gingival crevicular fluid (GCF) levels of inflammatory and bone remodelling related biomarkers following transplantation of a tissue-engineered biocomplex into intrabony defects at several time-points over 12-months”	“- Minimal Access Flap (MAF) surgical technique combined with a biocomplex of autologous clinical-grade alveolar bone-marrow mesenchymal stem cells in collagen scaffolds enriched with an autologous fibrin/platelet lysate - MAF surgery, with collagen scaffolds enriched with aFPL - MAF surgery alone”	“27 patients with remaining intrabony defects and periodontitis Stage III, Grade B/C, Age [20;68] years”	“- Levels of inflammatory and bone remodelling-related biomarkers in GCF were determined by ELISA - Collection of gingival crevicular fluid (GCF) from the osseous defects”	“At the protein level, the approach of MAF and biocomplex transplantation provided greater tissue regeneration potential as cell-based therapy appeared to modulate inflammation and bone remodelling in residual periodontal defects.”
Brizuela et al., 2024 [31]	“Revolutionizing Endodontics: Innovative Approaches for Treating Mature Teeth with Closed Apices and Apical Lesions: A Report of Two Cases”	Case reports	“Present the outcomes of 2 cases diagnosed with pulp necrosis and apical periodontitis in mature teeth treated with CB-RET”	“Cell-based RET (CB-RET) using encapsulated allogeneic umbilical cord mesenchymal stem cells (UC-MSCs) in a platelet-poor plasma (PPP)”	“2 patients with 50 and 43 year-old, both males”	“- Probing depths - CBCT diameter of radiolucency”	“This is the first study to report the success of an extended, 5-year follow-up for allogeneic CB-RET. This report presents an innovative and sustainable solution to challenging endodontic scenarios.”
Nakashima & Tanaka, 2024 [32]	“Therapy Using Pulp Regenerative Autologous Dental Pulp Stem Cells in a Mature Tooth with Apical Periodontitis: A Case Report”	Case reports	“Describe the potential utility of regenerative cell therapy in mature teeth with apical periodontitis”	“Autologous dental pulp stem cells (DPSCs)”	“44-year-old male patient”	“Cone-beam computed tomographic (CBCT) examinations”	“This case report demonstrated regeneration of pulp tissue containing sensory nerves using pulp regenerative therapy with autologous DPSCs in a mature tooth with apical periodontitis. The present technique might be useful in the field of endodontics by expanding the use of DPSCs to include mature teeth with posttreatment apical periodontitis to maintain pulpal function including dentin formation.”
Gomez-Sosa et al., 2024 [33]	“Allogeneic Bone Marrow Mesenchymal Stromal Cell Transplantation Induces Dentin Pulp Complex-like Formation in Immature Teeth with Pulp Necrosis and Apical Periodontitis”	Case-only observational clinical study	“Evaluated the capacity of allogeneic bone marrow MSCs (BM-MSCs) to regenerate pulp following necrosis and apical periodontitis in children’s permanent immature apex teeth.”	“allogeneic bone marrow MSCs (BM-MSCs)”	“14 patients of both sexes (8 male and 6 female) Age [8;12] years 15 teeth (13 incisors and 2 molars) with pulp necrosis and apical periodontitis”	“- Width of the apical foramen - Mineralization within the canal space - Sensitivity to cold and electricity - Clinical and radiographic evaluation of the periapical lesion”	“Transplantation of allogeneic MSCs induces the formation of dental pulp-like tissue in permanent immature apex teeth with pulp necrosis and apical periodontitis. Implant of MSCs constitutes a potential therapy in regenerative endodontics in pediatric dentistry. Future studies incorporating a larger sample size may confirm these results.”
Cubuk et al., 2023 [23]	“The effect of dental pulp stem cells and L-PRF when placed into the extraction sockets of impacted mandibular third molars on the periodontal status of adjacent second molars: a split-mouth, randomized, controlled clinical trial.”	Randomized controlled trial	“Compare the clinical and radiographic effectiveness of dental pulp stem cells (DPSCs) seeded onto L-PRF and L-PRF alone in the extraction socket of mandibular third molars”	“Dental pulp stem cells (DPSCs) with and without L-PRF”	“13 patients who required surgical removal of impacted bilateral mandibular third molars”	“- Probing pocket depth (PPD)—Clinical attachment levels (CAL)”	“This study found that there was a significant improvement regarding the PPD, CAL, and VD measurements with the application of L-PRF, both alone and with the addition of DPSC, at the extraction socket. DPSC did not significantly contribute to the results compared to L-PRF therapy alone.”
Elboraey et al., 2023 [24]	“Clinical and Radiographic Evaluation of Locally Delivered Plant Stem Cells for Treatment of Periodontitis: Randomized Clinical Trial”	Randomized controlled trial	“Evaluate clinically and radiographically the effectiveness of the local application of Edelweiss stem cells as a nonsurgical treatment for stage III periodontitis.”	“Edelweiss stem cells”	“40 periodontal pockets in participants who have stage III periodontitis Age [39;65] years”	“- Gingival index, - CAL - PPD - Bone density”	“Locally applied Edelweiss stem cells can be considered a promising nonsurgical treatment modality for periodontal regeneration.”
Gomez-Sosa et al., 2022 [34]	“Dental Pulp Regeneration Induced by Allogenic Mesenchymal Stromal Cell Transplantation in a Mature Tooth: A Case Report”	Case reports	“Capacity of allogeneic mesenchymal stromal cells (MSCs) to induce dental pulp and apical bone regeneration in a tooth previously endodontically treated.”	“Allogeneic mesenchymal stromal cells (MSCs)”	“55-year-old female patient consulting for swelling and a sinus tract associated with tooth #8”	“- Periapical bone density - Sensitivity to cold and electric pulp tests”	“This case report shows periodontal bone formation, apex remodeling, and dental pulp regeneration induced by allogeneic MSC transplantation in a mature nonvital tooth. Allogeneic MSCs may constitute a first-line therapy in regenerative endodontics”
Tobita et al., 2022 [25]	“Study protocol for periodontal tissue regeneration with a mixture of autologous adipose-derived stem cells and platelet rich plasma: A multicenter, randomized, open-label clinical trial”	“Multicenter, randomized, open-label comparative clinical trial”	“The aim of this study is to present the protocol of translation of tissue regeneration with ASCs and PRP into practical use, evaluating its efficacy.”	“Mixture of ASCs and PRP or enamel matrix derivate”	“15 patients will be randomly assigned to the treatment randomly divided into the ASCs and PRP transplantation group and the EMD group in a 2:1 ratio”	“- Height of alveolar bone before and after procedures”	“If effective, this cell therapy using autologous mesenchymal stem cells can represent a useful medical technology for regeneration of periodontal defects.”
Singhal et al., 2022 [26]	“A comparative evaluation of the effect of platelet rich fibrin matrix with and without peripheral blood mesenchymal stem cells on dental implant stability: A randomized controlled clinical trial”	Randomized controlled trial	“Compare and evaluate the effect of platelet rich fibrin matrix with and without peripheral blood mesenchymal stem cells on implant stability”	“Platelet rich fibrin matrix (PRFM) with and without peripheral blood mesenchymal stem cells (PBMSCs)”	“15 patients with 30 sites ensuring a minimum of two dental implants adjacently placed in an edentulous area; Age [25;50] years of both the sexes”	“- Bone to implant contact (BIC)”	“Platelet rich fibrin matrix and PBMSCs showed promising results as a potential regenerative material for increasing and enhancing BIC and hence implant stability.”
Apatzidou et al., 2021 [27]	“A tissue-engineered biocomplex for periodontal reconstruction. A proof-of-principle randomized clinical study”	Randomized controlled trial	“To assess the safety/efficacy of a tissue-engineered biocomplex in periodontal reconstruction.”	“- Autologous clinical-grade alveolar Bone-Marrow Mesenchymal-Stem- Cells (a-BMMSCs), seeded into collagen scaffolds, enriched with autologous fibrin/platelet lysate (aFPL) - Collagen scaffold/aFPL devoid of a-BMMSCs”	“Twenty-seven intrabony defects were block-randomized across three treatment-groups Age [20;86] years”	“- Clinical attachment - Pocket depth probing - Recession. - Radiographic Cemento-Enamel- Junction to - Bottom-Defect”	“Radiographic evidence of bone fill was less pronounced in Group-B, although clinical improvements were similar across groups. All approaches aimed to trigger the innate healing potential of tissues. Cell-based therapy is justified for periodontal reconstruction and remains promising in selected cases.”
Feng et al., 2021 [35]	“Small blood stem cells for enhancing early osseointegration formation on dental implants: a human phase I safety study”	Clinical trial	“Examine the safety and tolerability of SB cells in dental implantation for human patients with severe bone defects”	“Small blood stem cells (SB cells), isolated from human peripheral blood”	“9 patients were enrolled and divided into three groups with SB cell treatment doses Age [29;81] years $\bar{x}$ = 54 years Male to female ratio was 5:4”	“- Computed tomography (CT) scans to assess bone mineral density”	“This phase I study shows that treatment of SB cells for dental implantation is well tolerated with no major adverse effects. The use of SB cells for accelerating the osseointegration in high-risk dental implant patients warrants further phase II studies.”
Shin et al., 2020 [36]	“Reconstruction of Mandibular Defects with Bone Marrow-Derived Stem Cells in Odontogenic Myxoma”	Case reports	“Describes the process of mandibular reconstruction with autogenous bone graft, autologous human bone marrow mesenchymal stem cells (AHBM-MSCs), vertical distraction osteogenesis, dental implant installation, and prosthodontic treatment in a patient with odontogenic myxoma”	“Bone Marrow- Derived Stem Cells”	“A 54-year-old male patient”	/	“This case showed reconstruction of a mandibular defect caused by odontogenic myxoma. This study involved several procedures as the regeneration of new bone as therapeutic tools, including an autogenous bone graft, differentiated MSCs application, and ver- tical distraction osteogenesis. We hope further studies are needed to evaluate this procedure.”
Cordero et al., 2020 [37]	“Allogeneic Cellular Therapy in a Mature Tooth with Apical Periodontitis and Accidental Root Perforation: A Case Report”	Case reports	“Describe cell-based therapy using allogeneic umbilical cord mesenchymal stem cells (UC-MSCs) encapsulated in a bioscaffold for a complex case of a mature permanent tooth with apical periodontitis and accidental root perforation.”	“Allogeneic umbilical cord mesenchymal stem cells (UC-MSCs) encapsulated in a bioscaffold”	“19-year-old man undergoing orthodontic treatment was referred for endodontic treatment in tooth #7”	“- Periapical radiography, - Cone-beam computed tomographic imaging, - Sensitivity and vitality tests.”	“This case report reveals the potential use of allogeneic cellular therapy using encapsulated UC-MSCS in a platelet-poor plasma scaffold for a complex case of a permanent tooth with apical periodontitis and root perforation.”
Hernández-Monjaraz et al., 2020 [28]	“Dental Pulp Mesenchymal Stem Cells as a Treatment for Periodontal Disease in Older Adults”	“A quasi-experimental study”	“Determine the effectof a DPMSC treatment both the clinical improvement and regeneration of periodontic bone tissue and their relationship with the markers of chronic inflammation and oxidative stress of people in the aging process with PD”	“Collagen scaffold plus 5 × 106 of DPMSCs was placed by periodontal surgery. On the other hand, for the control group (CG), only collagen scaffolding without cells was placed”	“22 patients with PD was designed. Age [55;64] years All were volunteers, of both Sexes”	“- Bone mineral density (BMD) - Antioxidants Status (TAS), superoxide dismutase (SOD),lipoperoxides (LPO), and interleukins (IL) levels. - Depth of periodontal defect (DPD)”	“Our findings suggest that a DPMSCs treatment has an effect on periodontal bone regeneration in periodontal disease in aging people, linked to an increased superoxide dismutase, and decreased proinflammatory interleukins. Therefore, we conclude that a DPMSCs treatment can be a useful option to regenerate the lost tissues in periodontal disease.”
Abdal-Wahab et al., 2020 [29]	“Regenerative potential of cultured gingival fibroblasts in treatment of periodontal intrabony defects (randomized clinical and biochemical trial)”	“Randomized controlled trial”	“Clinically and biochemically investigate the use of gingival fibroblasts (GF) and their associated mesenchymal stem cells (GMSC) in the treatment of intrabony periodontal defects.”	“β-calcium triphosphate (β-TCP) followed by collagen membrane, cultured gingival fibroblasts and their associated mesenchymal stem cells (GMSC) and on the β-TCP scaffold and covered by a collagen membrane”	“20 patients (9 men and 11 females) having twenty peri odontal intrabony defect sites were randomly divided into two groups Age [32;50] years $\bar{x}$ = 43.4 ± 5.5 years”	“- Measurement of PDGF-BB and BMP-2 using the ELISA”	“Translocation of gingival fibroblasts from gingival tissue to periodontal defects could be a promising option that increases cellular elements with regeneration potential. The concept of total isolation of gingival fibroblasts using occlusive membranes must be re-evaluated”
Sánchez et al., 2020 [30]	“Periodontal regeneration using a xenogeneic bone substitute seeded with autologous periodontal ligament-derived mesenchymal stem cells: A 12-month quasi-randomized controlled pilot clinical trial”	“Randomized controlled trial”	“Evaluate the safety and efficacy of autologous periodontal ligament-derived mesenchymal stem cells (PDL-MSCs) embedded in a xenogeneic bone substitute (XBS) for the regenerative treatment of intra-bony periodontal defects”	“Autologous periodontal ligament-derived mesenchymal stem cells (PDL-MSCs) embedded in a xenogeneic bone substitute (XBS)”	“20 patients: Control group (n = 10) Age [38;60] years $\bar{x}$ = 48.8 (SD = 10.6) Male to female ratio was 7:3 Test group (n = 10) Age [49;65] years $\bar{x}$ = 57,5 (SD = 7.9) Male to female ratio was 7:3”	“- Clinical and radiographical parameters - Clinical attachment level (CAL) - Probing pocket depth (PPD)”	“The application of PDL-MSCs to XBS for the treatment of one- to two-wall intra-bony lesions was safe and resulted in low postoperative morbidity and appropriate healing, although its additional benefit, when compared with the XBS alone, was not demonstrated”

### 3.3. Overall Interpretation of Quantitative Outcomes

Of the 12 reported outcomes, 10 favored stem cell-based interventions with statistically significant improvements (*p* < 0.05), while 2 outcomes showed comparable results between stem cell and control groups. Mean differences consistently demonstrated clinically meaningful improvements in the stem cell groups, including: Defect fill percentage, CAL improvement, Radiographic bone gain and Bone mineral density, the results are presented in Table 2.

**Table 2:** Key Quantitative Findings Across Included Trials.

Study	Design	Intervention	Comparator	Primary Outcome	Test Group Result	Control Group Result	Mean Difference	Follow-up (Months)	Direction	Statistical Significance
Sreeparvathy et al., 2024 [21]	RCT (split-mouth)	PRFM + PBMSCs	PRFM alone	Defect fill %	63.52 ± 14.86%	45.20 ± 15.95%	+18.32%	6	Favors SC	*p* < 0.001
Sreeparvathy et al., 2024 [21]	RCT (split-mouth)	PRFM + PBMSCs	PRFM alone	CAL (mm)	4.24 ± 0.83	5.12 ± 0.93	−0.88 mm	6	Favors SC	*p* < 0.001
Apatzidou et al., 2024 [22]	RCT (3-arm)	MAF + a-BMMSCs + aFPL	MAF + aFPL/MAF alone	CEJ-BD reduction (mm)	5.1 ± 2.8	7.7 ± 2.2/6.8 ± 1.9	Superior bone fill	12	Favors SC	*p* < 0.001
Elboraey et al., 2023 [24]	RCT	SRP + plant stem cells	SRP alone	PPD (mm)	3.25 ± 0.55	4.1 ± 0.3	−0.85 mm	3	Favors SC	*p* = 0.006
Elboraey et al., 2023 [24]	RCT	SRP + plant stem cells	SRP alone	Bone mineral density	143.92 ± 9.96	108 ± 13.23	+35.92 units	6	Favors SC	*p* = 0.008
Cubuk et al., 2023 [23]	RCT (split-mouth)	L-PRF + DPSC	L-PRF alone	CAL reduction (mm)	2.12 ± 0.74	2.23 ± 1.45	No difference	6	Comparable	*p* > 0.05
Singhal et al., 2022 [26]	RCT	PRFM + PBMSCs	PRFM alone	ISQ at 3 months	79.00 ± 2.07	74.60 ± 2.95	+4.4 units	3	Favors SC	*p* = 0.001
Apatzidou et al., 2021 [27]	RCT (3-arm)	a-BMMSCs + collagen + aFPL	Collagen + aFPL/MAF alone	CEJ-BD reduction (mm)	2.1 (1.4–2.8)	0.1 (0.0–0.7)/1.3 (1.0–1.8)	+2.0 mm vs. control B	12	Favors SC	*p* = 0.001
Hernández-Monjaraz et al., 2020 [28]	Quasi-experimental	Collagen + DPMSC	Collagen alone	Defect depth reduction (mm)	3.32 ± 0.12	1.80 ± 0.15	+1.52 mm	6	Favors SC	*p* = 0.001
Abdal-Wahab et al., 2020 [29]	RCT	β-TCP + GMSC	β-TCP alone	VPD reduction (mm)	3.10 ± 0.88	5.20 ± 0.80	−2.10 mm	6	Favors SC	*p* < 0.0001
Abdal-Wahab et al., 2020 [29]	RCT	β-TCP + GMSC	β-TCP alone	Bone gain (mm)	3.14 ± 1.33	1.91 ± 0.16	+1.23 mm	6	Favors SC	*p* < 0.0001
Sánchez et al., 2020 [30]	Quasi-RCT	XBS + PDL-MSCs	XBS alone	CAL gain (mm)	1.44 ± 1.87	0.80 ± 1.68	+0.64 mm	12	Comparable	*p* > 0.05

### 3.4. Sample Characterization for Study Quality Assessment

The quality assessments are summarized in Table 3 for the randomized controlled trials and in Table 4 for the case reports. The evaluation was based on the number of ‘YES’ responses obtained for each article, providing an objective measure of quality. As a result, ten articles [21,24,26,27,30,31,32,34,35,37] were classified as ‘good’ quality/low bias, while seven studies [22,23,25,28,29,33,36] demonstrated moderate evidence/moderate bias.

**Table 3:** Joanna Briggs Institute Critical Appraisal Checklist for Randomized Controlled Trials.

Joanna Briggs Institute Critical Appraisal Checklist for Randomized Controlled Trials. [20]	“1. Was True Randomiza- tion Used for Assignment of Participants to Treatment Groups ?”	“2. Was Allocation to Treatment Groups Concealed?”	“3. Were Treatment Groups Similar at the Baseline?”	“4. Were Participants Blind to Treatment Assignment ?”	“5. Were Those Delivering Treatment Blind to Treatment Assignment ?”	“6. Were Outcomes Assessors Blind to Treatment Assignment ?”	“7. Were Treatment Groups Treated Identically Other than the Intervention of Interest ?”	“8. Was Follow up Complete and If Not, Were Differences Between Groups in Terms of Their Follow up Adequately Described and Analyzed ?”	“9. Were Participants Analyzed in the Groups to Which They Were Random- ized?”	“10. Were Outcomes Measured in the Same Way for Treatment Groups ?”	“11. Were Outcomes Measured in a Reliable Way ?”	“12. Was Appropriate Statistical Analysis Used ?”	“13. Was the Trial Design Appropriate, and Any Deviations from the Standard RCT Design (Individual Randomiza- tion, Parallel Groups) Accounted for in the Conduct and Analysis of the Trial ?”
Sreeparvathy et al., 2024 [21]	Y	Y	Y	Y	Y	Y	UN	Y	UN	Y	Y	Y	UN
Cubuk et al., 2023 [23]	Y	UN	Y	N	N	Y	Y	Y	Y	Y	Y	Y	Y
Apatzidou et al., 2021 [27]	Y	Y	Y	Y	Y	Y	Y	Y	Y	Y	Y	Y	Y
Sánchez et al., 2020 [30]	UN	UN	Y	Y	Y	Y	Y	Y	Y	Y	Y	Y	UN
Abdal-Wahab et al., 2020 [29]	Y	UN	Y	N	N	Y	Y	Y	Y	Y	Y	Y	Y
Tobita et al., 2022 [25]	Y	N	Y	N	N	N	Y	Y	Y	Y	Y	Y	Y
Elboraey et al., 2023 [24]	Y	UN	Y	UN	UN	UN	Y	Y	Y	Y	Y	Y	Y
Singhal et al., 2022 [26]	Y	Y	Y	Y	N	Y	Y	Y	Y	Y	Y	Y	Y
Apatzidou et al., 2024 [22]	Y	UN	Y	N	N	Y	Y	Y	Y	Y	Y	Y	Y
Hernández-Monjaraz et al., 2020 [28]	Y	UN	Y	UN	UN	UN	Y	Y	Y	Y	Y	Y	N

**Table 4:** Joanna Briggs Institute Critical Appraisal Checklist for Case reports.

Joanna Briggs Institute Critical Appraisal Checklist for Case Reports. [20]	“1. Were the Patient’s Demographic Characteristics Clearly Described?”	“2. Was the Patient’s History Clearly Described and Presented as a Timeline?”	“3. Was the Current Clinical Condition of the Patient on Presentation Clearly Described?”	“4. Were Diagnostic tests or Assessment Methods and the Results Clearly Described?”	“5. Was the Intervention(s) or Treatment Procedure(s) Clearly Described?”	“6. Was the Post-intervention Clinical Condition Clearly Described?”	7. Were Adverse Events (Harms) or Unanticipated Events Identified and Described?	“8. Does the Case Report Provide Takeaway Lessons?”
Cordero et al., 2020 [37]	Y	Y	Y	Y	Y	Y	N	Y
Shin et al., 2020 [36]	UN	Y	Y	Y	Y	Y	N	Y
Gomez-Sosa et al., 2022 [34]	Y	Y	Y	Y	Y	Y	N	Y
Nakashima & Tanaka, 2024 [32]	Y	Y	Y	Y	Y	Y	UN	Y
Brizuela et al., 2024 [31]	Y	Y	Y	Y	Y	Y	UN	Y
S Feng et al., 2021 [35]	Y	Y	Y	Y	Y	Y	Y	Y
Gomez-Sosa et al., 2024 [33]	N	N	Y	Y	Y	Y	N	Y

### 3.5. Types of Treatments

Overall, 17 articles comprising 245 patients were included in this study, in which different types of stem cell-based therapies were applied. with different origins such as periodontal ligament, Fibroblast, Dental Pulp, Allogeneic umbilical cord, Bone Marrow, Small blood, Alveolar Bone, Peripheral blood, Adipose-derived, Stromal cells, Edelweiss; also in combination with various elements like Platelet Rich Fibrin Matrix, Platelet-poor plasma, enamel matrix derivative, Fibrin/platelet lysate (aFPL), Collagen scaffold, β-calcium triphosphate (β-TCP) followed by collagen membrane, cultured gingival fibroblasts and Xenogeneic bone substitute (XBS). MAF surgery was also combined with cellular therapy. (Table 1. for additional details).

### 3.6. Outcome Measurements

Biochemistry test, clinical and radiographic measurements were employed to assess various parameters such as recessions, probing pocket depth, periodontal defect depth clinical attachment loss and level, levels of Platelet-derived growth factor B subunits (PDGF-BB) and Human Bone Morphogenetic Protein-2 (BMP-2), Antioxidants Status levels (superoxide dismutase, lipoperoxides, interleukins), Height of alveolar bone, bone mineral density, diameter of periapical bone radiolucency, cemento enamel junction status, bottom defect, Sensitivity and vitality tests, bone to implant contact and collection of gingival crevicular fluid. (Table 1. for additional details).

## 4. Discussion

Stem cell-based periodontal regeneration shows benefits, but outcomes depend on defect type, scaffold or adjunct, cell source, and follow-up length [17,21,23,27,30]. Across small randomized and controlled trials, clinical attachment gain and radiographic bone fill are reported, yet effect sizes vary and follow up is often limited to twelve months in most studies [21,23,27,30]. Variability likely stems from differences in harvesting and processing, In Vitro expansion, storage and dose, and the choice of scaffold, such as PDLSCs on xenograft, PBMSCs with PRF, or bio-complex constructs [21,27,30]. To improve reproducibility and translation, future studies should standardize cell characterization and dosing, detail scaffold properties, use common outcome sets, and extend follow up, as outlined in a recent multicenter protocol [25].

### 4.1. Cell Culture Techniques

Cell culture techniques play a crucial role in determining clinical outcomes. The protocols involved must be rigorously standardized and tightly controlled. In the studies reviewed, the isolation and culture techniques varied considerably across the different protocols.

#### 4.1.1. Expansion

Differences between studies often reflect how cells are processed. Key variables include isolation method, culture conditions, passage number, and the use of scaffolds or platelet derivatives [18,21,27,30]. Most periodontal protocols rely on ex vivo expansion to reach a target cell dose, for example, PDLSCs seeded on scaffolds or assembled into bio-complex constructs [27,30], whereas others deliver minimally manipulated preparations or pursue in situ recruitment with platelet products [21,25,38]. Evidence comparing these strategies head-to-head is limited, but trials with ex vivo expanded constructs have reported gains in clinical attachment and radiographic bone fill over controls, suggesting a potential advantage that requires confirmation in larger studies [27]. Given the heterogeneity, studies should report dose, passage and viability, detail culture parameters, and justify when expansion is omitted or replaced by recruitment approaches [18,25].

#### 4.1.2. Growth Medium

In the included studies, culture media were chosen with clinical feasibility in mind, favoring α-MEM combined with autologous supplements rather than animal serum when cells were prepared for transplantation [27,34]. This choice aligns with translational considerations and with protocols that pair cells with platelet derivatives at surgery, reinforcing a paracrine-oriented strategy [21,25,27].

#### 4.1.3. Culture Conditions

Culture conditions can shape paracrine activity, including Vascular Endothelial Growth Factor (VEGF) secretion. In dental pulp models, hypoxia increases VEGF-A and supports angiogenesis, suggesting a potential benefit for regenerative goals, although the clinical signals in our set are indirect and largely endodontic [33,39]. Under hypoxic conditions, reduced oxygen availability stabilizes hypoxia-inducible factor-1α (HIF-1α) by preventing prolyl hydroxylase domain (PHD)-mediated hydroxylation and subsequent von Hippel–Lindau (VHL)-dependent proteasomal degradation. Accumulated HIF-1α translocates to the nucleus, heterodimerizes with HIF-1β, and activates transcription of VEGF and other pro-angiogenic genes Via hypoxia-response elements. In dental pulp stem cells, this pathway also enhances osteogenic differentiation markers and upregulates CXCR4 expression, facilitating stem cell homing through the SDF-1/CXCR4 axis [40]. Some protocols likely incorporate reduced oxygen during expansion, but detailed reporting is inconsistent, so we cannot determine its added value in periodontitis [32,33]. Because VEGF and BMP-2 also promote bone regeneration and stem-cell homing, testing hypoxia culture against normoxia in periodontal lesions is reasonable, but it remains a hypothesis that requires controlled trials [39]. Independently of oxygen tension, rigorous cell identity and potency testing should be standard, with positive MSC markers (e.g., CD29, CD73, CD90…) negative hematopoietic markers (CD34/CD45), and osteogenic assays reported, as exemplified by the periodontal biocomplex trial [27] using flow cytometry and differentiation testing [32,34].

### 4.2. Cell Provenance and the Impact of Efficacy

Choosing the cell source should follow the defect to be treated, the delivery scaffold, and the practical workflow. For intrabony periodontal defects, in a single randomized clinical trial [21], peripheral blood mesenchymal stem cells delivered with PRF-type matrices showed clinically promising defect fill. A separate randomized study reported improved implant stability when PBMSCs were used around implants, suggesting a potential benefit [26]. For engineered reconstruction, in one trial, an a-BMMSC bio-complex graft was feasible but showed comparable results to minimally invasive surgery at twelve months for CAL and PPD, so BM-MSC bio-complex grafts are best positioned when larger or composite defects justify a complex graft, a situation also illustrated in craniofacial reconstruction reports [27,36]. DPSC-based approaches produced variable results. Adding DPSCs to L-PRF did not improve socket healing outcomes in a split-mouth trial, and densitometric gains in older adults were not statistically significant at six months [23,28]. Periodontal ligament stem cells seeded on a xenogeneic scaffold are feasible in periodontitis, with early signals in a small quasi randomized pilot, but confirmation in larger trials is needed before broad adoption [30]. Outside strict periodontitis, case-based evidence shows that bone marrow or pulp derived cells can support dentin pulp complex formation, which is relevant mainly to endodontic indications rather than periodontal defects [33,34]. Small blood stem cells have yielded early osseointegration signals in a phase I study, suggesting a role in implant contexts rather than periodontal intrabony lesions [35]. From a workflow perspective, DPSC and PDLSC approaches typically require enzymatic digestion and multi pass expansion, increasing time and Good Manufacturing Practice (GMP) requirements, whereas PBMSC products can be prepared closer to the point of care and fit same session or short interval protocols [21,26].

### 4.3. Factors Influencing Osteogenic Differentiation and Role of Stem-Cells

Across the included trials, pairing cells with a biologically active carrier strengthened early periodontal outcomes when the carrier already had clinical utility. In one randomized trial of intrabony defects, PRFM combined with PBMSCs outperformed PRFM alone at three to six months for early defect fill and for CAL and PPD, consistent with a synergistic scaffold-plus-cell effect [21]. In a split-mouth trial at extraction sockets, L-PRF improved PPD, CAL, and radiographic density, but adding DPSCs did not confer additional benefit over L-PRF alone, so routine DPSC augmentation at sockets is not supported by current evidence [23]. In craniofacial reconstruction, adding BM-MSCs to PRP shortened consolidation time compared with PRP alone, a finding that supports a role for cells in accelerating hard-tissue maturation when large bony spans are involved [36]. At the mechanistic level, patients treated with an autologous MSC biocomplex showed increases in IL-10 and BMP-7 with concurrent reductions in TNF-α and RANKL and a favorable RANKL/OPG shift, which is consistent with paracrine immune modulation that supports bone formation [22].

In one trial using β-TCP carriers, adding cultured gingival fibroblasts improved vertical pocket depth and clinical attachment versus β-TCP alone, indicating that even non-stem stromal cells can contribute to a pro-regenerative environment when anchored in a suitable scaffold [29]. In older adults with periodontitis, DPSC-based therapy produced only modest densitometric change at six months, so any osteogenic advantage appears context-dependent and likely relies on an effective carrier and defect containment [28]. The clinical trials are, first, use a scaffold or matrix with documented clinical benefit and then consider adding cells when a faster or larger early response is needed [21,23], second, expect the clearest gains when the defect is accessible to stable matrix placement and when paracrine support can be captured by the carrier [21,22,29], and third, reserve cell-augmented strategies for indications where the added procedural steps are justified by the clinical goal, such as multi-wall intrabony lesions or segmental bony spans [21,36].

### 4.4. Impact of Number of Transplanted Cells

In a study comparing three groups receiving increasing doses of Small Blood Stem Cells (SBSCs) (1 × 10^5^, 1 × 10^6^, and 1 × 10^7^ cells) [35], the group that received the highest quantity of SBSCs demonstrated a trend towards accelerated improvement in bone density, although this difference was not statistically significant compared to the lower-dose groups [35]. This preliminary finding suggests that the number of implanted cells might influence healing kinetics, likely due to a paracrine threshold effect, and larger trials are needed to confirm a dose-response relationship and determine the optimal quantity for clinical efficacy [35].

### 4.5. Type of Regenerated Lesions and Different Locations

#### 4.5.1. Intrabony Periodontal Defects

In intrabony defects, pairing a biologically active matrix with cells improves early healing; for example, the “Supercell” protocol showed greater percent defect fill at six months than the same matrix alone [21]. With β-TCP carriers, adding cultured gingival fibroblasts led to larger clinical gains than β-TCP alone at six months, supporting the idea that stromal cell augmentation is helpful in contained periodontal defects [29]. By contrast, when disease presents as more diffuse horizontal loss, responses are slower and smaller, as seen with dental pulp cell therapy in older adults where bone density rose modestly over six months and did not reach statistical significance [28]. Practically, cell-augmented matrices should be favored for localized, well-vascularized defects, with slower changes anticipated in diffuse lesions.

#### 4.5.2. Periapical Bone Lesions Associated with Endodontic Pathology

Cell-based regenerative endodontic therapy (CB-RET) using allogeneic or autologous MSCs demonstrated successful periapical bone healing and pulp-like tissue formation in mature and immature teeth with apical periodontitis [31,32,33,34,37]. Long-term follow-up (up to 5 years) showed sustained sensory function and continued dentin formation. CB-RET represents a paradigm shift from traditional root canal therapy toward biological regeneration, although these findings are derived exclusively from case reports and require confirmation through randomized controlled trials.

#### 4.5.3. Peri-Implant Bone and Craniofacial Reconstruction

In one implant study, PBMSCs improved stability compared with PRFM alone, with significant ISQ separation evident by one month [26], and small blood stem cell protocols reported early shifts toward denser bone quality over twelve weeks based on CT assessments [35]. In large segmental mandibular defects, multimodal regenerative approaches combining autogenous bone, BM-MSCs, and distraction osteogenesis facilitated bone regeneration sufficient for implant placement. Expect early mechanical benefits around implants when PBMSCs are used with PRF-type carriers [26], and reserve complex cell-augmented strategies for extensive craniofacial defects where the added procedural steps are justified by the clinical goal.

### 4.6. Safety

Across the included clinical studies, stem cell interventions were well tolerated, with no treatment related serious adverse events reported and only expected local effects like transient swelling or postoperative sensitivity when noted [21,23,26,27,32,35]. Reports using allogeneic bone marrow-derived cells did not describe immune rejection or hypersensitivity, consistent with the clinical immunomodulatory profile observed for these products [33,34,37].

### 4.7. Regulatory Considerations

The clinical translation of stem cell-based periodontal therapies is constrained by regulatory challenges. In the European Union, these therapies are classified as Advanced Therapy Medicinal Products (ATMPs) under Regulation (EC) N° 1394/2007, specifically as Somatic Cell Therapy Medicinal Products (SCTMPs) or Tissue-Engineered Products (TEPs), depending on the degree of cell manipulation and scaffold integration. In the United States, the FDA regulates them as Biological Products under Section 351 of the Public Health Service Act, requiring Investigational New Drug (IND) applications for clinical trials and Biologics License Applications (BLA) for market authorization. As of 2025, no stem cell-based periodontal therapy has received full marketing authorization from either the EMA or FDA for routine clinical use. Barriers to approval include lack of standardized manufacturing protocols, high GMP compliance costs, variable clinical outcomes, and limited long-term safety data. Future development should prioritize early regulatory engagement, multi-center randomized trials with extended follow-up, and exploration of off-the-shelf allogeneic products to reduce complexity and cost.

### 4.8. Economic and Clinical Practice Implications:

#### 4.8.1. Economic Considerations and Cost-Effectiveness

A critical limitation is the absence of cost-effectiveness analyses in all 17 included studies. None reported treatment costs or economic outcomes. Manufacturing costs for ATMP-classified stem cell therapies under GMP compliance are substantial, with estimates ranging from €5.6M to €9.9M [41] annually for large-scale production and $5000–$50,000 per patient for autologous expanded cell products. Key cost drivers include cell isolation and expansion, quality control testing, regulatory compliance, scaffold materials, and surgical delivery. Conventional periodontal surgeries cost $1500–$5000 per site, making stem cell therapies 2–10 times more expensive initially, though long-term cost-effectiveness remains unclear. Strategies to reduce costs include minimally manipulated point-of-care protocols, off-the-shelf allogeneic products, automation, and outcome-based pricing. Future trials must incorporate formal economic evaluations (direct/indirect costs, cost-effectiveness ratios, budget impact analyses) to inform clinical adoption and reimbursement decisions.

#### 4.8.2. Implications for Clinical Practice

Based on this systematic review and current evidence, here are the key points guiding when to consider cell therapy versus conventional approaches for periodontal bone lesions. While cell therapy in periodontology remains mostly investigational, it may be considered for select cases with careful patient selection.

### 4.9. Decision Framework: Conventional Treatment or Stem Cell/Regenerative Approaches

#### 4.9.1. Indications Favoring Conventional Therapy

For periodontal bone lesions, conventional therapy is particularly indicated in the following scenarios: when defects are characterized by single or double walls, or present as horizontal bone loss with shallow or broad lesions. Patients who have poor oral hygiene or demonstrate unwillingness to comply with required postoperative care, as well as those with uncontrolled systemic risk factors such as diabetes or a smoking habit exceeding ten cigarettes per day, are also best managed using traditional approaches. Additionally, conventional treatment should be favored in cases where financial cost or logistical complexity restricts access to more advanced interventions. In these situations, established modalities like open flap debridement, guided tissue regeneration with biomaterials, bone grafts, and platelet-rich fibrin or plasma are well supported by long-term clinical evidence and consistently yield effective, predictable outcomes.

#### 4.9.2. Indications Favoring Cell Therapy or Regenerative Cell-Based Approaches

Cell therapy may be considered for periodontal bone lesions in specific clinical situations. These include deep, well-contained (2–3 wall) intrabony defects greater than 3 mm, particularly where conventional regenerative therapy has failed or reached a plateau. This approach is also relevant for large periodontal, and for grade II furcation defects that have not responded to standard guided tissue regeneration techniques. Patients who are young, systemically healthy, highly motivated, and maintain optimal oral hygiene may be suitable candidates for more advanced, investigational cell-based therapies. In all cases, the patient should be well informed regarding the experimental status of cell therapy, associated costs, and the necessity for close post-operative monitoring.

## 5. Conclusions

The evidence from this systematic review suggests that stem cell-based methods demonstrate greater regenerative potential than traditional methods in the management of periodontal bone lesions. This benefit is largely due to their capacity to induce cellular differentiation, support angiogenesis, and modulate the inflammation, by downregulating proinflammatory cytokines (e.g., TNF-α, IL-6) and controlling endothelial permeability, which in turn promotes tissue regeneration. A comparison between the 17 included studies highlighted an overall positive signal of efficacy for cell-based tissue engineering strategies. Moreover, clinical safety seems to be good, and no serious complications have been reported. The mechanisms involve immune modulation and osteogenesis. Although promising, the effectiveness observed remains contingent upon several critical factors, such as the source of the cells, culture protocols, associated biomaterials, and the overall quality of the experimental design, including the type of defect, surgical approach, and cell density. Moreover, the implementation of these therapies continues to face notable technical, economic, and regulatory challenges that hinder their widespread adoption in routine clinical practice. High costs and complex administrative procedures also represent significant barriers. While the prospects remain highly promising, the successful integration of these therapies into everyday clinical practice will depend on the completion of large-scale, long-term clinical trials. Such studies will be crucial to develop simpler, standardized, and more affordable protocols and truly assess the reproducibility of these treatments.

## Data Availability

No new data were generated or analyzed in this study. Data sharing is not applicable to this article as it is based on a systematic review of the publicly available literature.

## Supplementary Materials

The following supporting information can be downloaded at: https://www.mdpi.com/article/10.3390/clinpract15120222/s1: PRISMA 2020 checklist.

## Abbreviations

**Abbreviation**	**Full Term**		
a-BMMSC	Autologous Bone Marrow Mesenchymal Stem Cell	PBMSC	Peripheral Blood Mesenchymal Stem Cell
aFPL	Autologous Fibrin/Platelet Lysate	PDGF-BB	Platelet-Derived Growth Factor-BB
α-MEM	Alpha Minimum Essential Medium	PDLSC	Periodontal Ligament Stem Cell
BMP-2	Bone Morphogenetic Protein-2	PDL-MSC	Periodontal Ligament-Derived Mesenchymal Stem Cell
CAL	Clinical Attachment Level	PPD	Probing Pocket Depth
CBCT	Cone Beam Computed Tomography	PRF	Platelet-Rich Fibrin
DPD	Depth of Periodontal Defect	PRFM	Platelet-Rich Fibrin Matrix
DPSC	Dental Pulp Stem Cell	RANKL	Receptor Activator of Nuclear Factor Kappa-Β Ligand
ELISA	Enzyme-Linked Immunosorbent Assay	RCT	Randomized Controlled trial/Randomized Clinical trial
GCF	Gingival Crevicular Fluid	SBSC	Small Blood Stem Cell
GF	Gingival Fibroblast	SC	Stem Cell
GMSC	Gingival Mesenchymal Stem Cell	SRP	Scaling and Root Planing
IL	Interleukin	TNF-α	Tumor Necrosis Factor Alpha
ISQ	Implant Stability Quotient	VEGF	Vascular Endothelial Growth Factor
L-PRF	Leukocyte-Platelet Rich Fibrin	XBS	Xenogeneic Bone Substitute
MAF	Minimal Access Flap	β-TCP	Beta-Tricalcium Phosphate
MSC	Mesenchymal Stem Cell		
